# Race, Ethnicity, and Immigration Status in a Medical Licensing Educational Resource: a Systematic, Mixed-Methods Analysis

**DOI:** 10.1007/s11606-021-06843-0

**Published:** 2021-05-13

**Authors:** Jessica P. Cerdeña, Rohit Jaswaney, Marie V. Plaisime

**Affiliations:** 1 Yale School of Medicine, New Haven, CT USA; 2 Department of Anthropology, Yale University, New Haven, CT USA; 3 New York Medical College, Valhalla, NY USA; 4 Department of Sociology, Howard University, Washington, DC USA

**Keywords:** USMLE, race-based medicine, race, ethnicity, immigration status

## Abstract

**Background:**

Medical students preparing for the United States Medical Licensing Exam (USMLE) Step 2 Clinical Knowledge (CK) Exam frequently use the UWorld Step 2 CK Question Bank (QBank). Over 90% of medical students use UWorld QBanks to prepare for at least one USMLE. Although several questions in the QBank mention race, ethnicity, or immigration status, their contributions to the QBank remain underexamined.

**Objective:**

We conducted a systematic, mixed-methods content analysis to assess whether and how disease conditions might be racialized throughout this popular medical education resource.

**Design:**

We screened 3537 questions in the QBank between May 28 and August 11, 2020, for mentions of race, ethnicity, or immigration status. We performed multinomial logistic regression to assess the likelihood of each racial/ethnic category occurring in either the question stem, answer explanation, or both. We used an inductive technique for codebook development and determined code frequencies.

**Main Measures:**

We reviewed the frequency and distribution of race or ethnicity in question stems, answer choices, and answer explanations; assessed associations between disease conditions and racial and ethnic categories; and identified whether and how these associations correspond to race-, ethnicity-, or migration-based care.

**Results:**

References to Black race occurred most frequently, followed by Asian, White, and Latinx groups. Mentions of race/ethnicity varied significantly by location in the question: Asian race had 6.40 times greater odds of occurring in the answer explanation only (95% CI 1.19–34.49; *p* < 0.031) and White race had 9.88 times greater odds of occurring only in the question stem (95% CI 2.56–38.08; *p* < 0.001). Qualitative analyses suggest frequent associations between disease conditions and racial, ethnic, and immigration categories, which often carry implicit or explicit biological and genetic explanations.

**Conclusions:**

Our analysis reveals patterns of race-based disease associations that have potential for systematic harm, including promoting incorrect race-based associations and upholding cultural conventions of White bodies as normative.

**Supplementary Information:**

The online version contains supplementary material available at 10.1007/s11606-021-06843-0.

## INTRODUCTION

Biomedical researchers frequently deploy the terms “race” and “ethnicity” to describe study populations. Although scientists often treat race as an essential, biological variable, race is a complex sociopolitical construct that shapes relations between individuals and groups.^1,2^ Race-based medicine involves the misuse of biological understandings of race in ways that promote unequal treatment.^3^ Despite extensive scholarship demonstrating how beliefs in inherent racial differences reinforce racial health inequities,^3,4^ medical training often frames race as an independent risk factor for disease, suggesting that race serves a biological function.^5,6^

Though medical training varies across the USA, medical students rely on standardized study materials for the United States Medical Licensing Examinations (USMLEs).^7,8^ The USMLE World (UWorld) Question Bank (QBank) is a widely used tool containing USMLE-style practice questions. Over 90% of medical students report using UWorld for at least one USMLE.^9^ Studies indicate that “one-step” clinical associations produce high success rates in self-directed retrieval-studying.^10^ Reliance on heuristics involving flawed race-based disease associations risks reinscribing harmful, biologized notions of race.^5^ Despite extensive use of study resources such as the UWorld Step 2 Clinical Knowledge (CK) QBank, the educational function of mentions of race, ethnicity, and immigration status remains poorly understood. This cross-sectional study examines whether and how race-based medicine operates throughout the UWorld Step 2 CK QBank by (1) reviewing the frequency and distribution of race, ethnicity, and immigration status in question stems, answer choices, and answer explanations; (2) assessing associations between disease conditions and racial, ethnic, and immigration categories; (3) evaluating biological explanations provided; and (4) identifying whether and how these associations correspond to race-, ethnicity-, or migration-based care.

## METHOD

### Data Source

We examined all 3537 available questions in the UWorld Step 2 CK QBank between May 28 and August 11, 2020. We identified and analyzed 123 questions (3.5%) in which race, ethnicity, nationality, or immigration status was included in the question stem or answer explanation. We extracted racial/ethnic or immigration status identifiers, the surrounding text, the two most common answer choices, and the percentage of users who chose the two most common answers in Google sheets. We simplified racial/ethnic categories to roughly align with Census definitions with the additions of “Ashkenazi Jewish,” “Mediterranean,” and “Middle Eastern.”^11^ We simplified migration origins to approximate World Bank regions,^12^ with just one Asian region. We excluded mentions of immigration status that included references to narrow areas in which specific infectious diseases are endemic in the answer explanation from quantitative analyses but retained these in qualitative analyses. The Yale University Institutional Review Board determined that this study did not constitute human subjects research.

### Content Analysis and Codebook Development

We developed our codebook using an inductive technique. Two of us as coders (JPC and RJ) independently reviewed the data and proposed preliminary codes. JPC and RJ compared codes from the initial review and settled on 15 codes (see Appendix for full codebook). After systematically applying these 15 codes to the data (κ = 0.64), JPC and RJ discussed and refined the codebook to clarify definitions; a third coder (MVP) resolved discrepancies to prepare the dataset for analysis.

### Analysis

We used Pearson’s *χ*^2^ analyses to identify differences by race/ethnicity among question locations and UWorld systems. We then used multinomial logistic regression to assess the likelihood of each racial/ethnic category occurring in either the question stem, answer explanation, or both. To avoid non-convergence of the model, we excluded racial/ethnic categories that did not occur in the question stem. JPC conducted statistical analyses in RStudio, version 1.1.463 (RStudio PBC, Boston, MA). We calculated frequencies for each code and developed these into the themes relating to our research questions.

## RESULTS

### Frequency and Distribution of Race, Ethnicity, and Immigration Status

Eighty-three questions mentioned racial/ethnic categories. Mentions of Black race occurred most often (*n* = 49), then Asian (*n* = 23), White (*n* = 22), and Latinx (*n* = 15) groups (see Table 1). References to immigration status occurred in 40 questions. Of these, eight referenced specific areas associated with infectious disease risk and were excluded from quantitative analysis. Among the other 32, Asian origin occurred most frequently (*n* = 11), followed by European (*n* = 7) and sub-Saharan African (*n* = 4).

**Table 1:** Prevalence of Racial/Ethnic Categories and Immigration Regions of Origin

**Race/ethnicity**		**Immigration Status**	
**Category**	**Mentions (*n*, %)**	**Category**	**Mentions (*n*, %)**
Ashkenazi Jewish	2 (1.4)	Asia	11 (34.4)
Asian	23 (15.8)	Europe	7 (21.9%)
Black	59 (40.4)	Latin America	3 (9.4%)
Latinx	15 (10.3)	Middle East and North Africa	3 (9.4%)
Mediterranean	4 (2.7)	sub-Saharan Africa	4 (12.5%)
Middle Eastern	7 (4.8)	Unspecified	4 (12.5%)
Native	5 (3.4)		
White	22 (15.1)		
Other	9 (6.2)		

**Figure 1:**
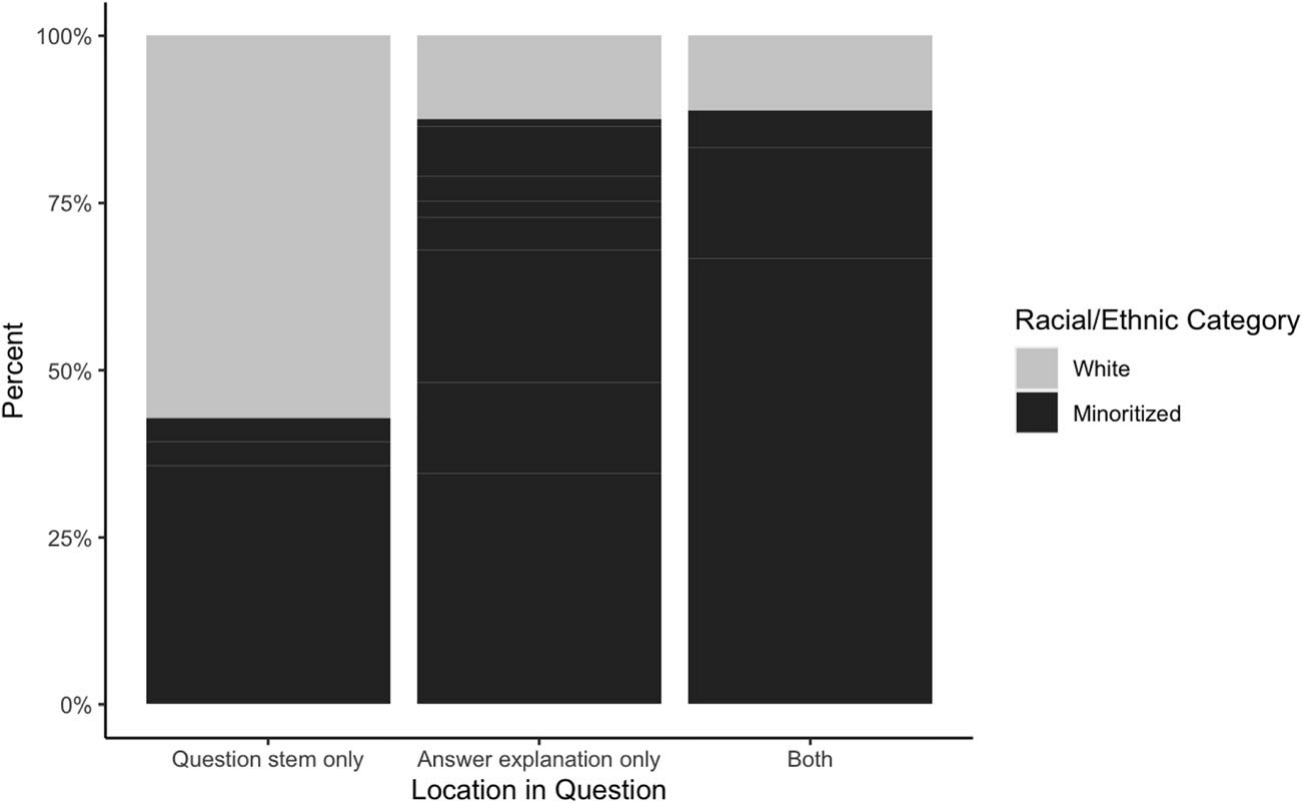
Race/ethnicity by location in question. This stacked bar plot represents mentions of racial/ethnic categories by location in the question (i.e., question stem only, answer explanation only, or both question stem and answer explanation). Racial/ethnic categories are represented as percentages of questions that mentioned race/ethnicity. “Minoritized” includes “Black,” “Latinx,” “Asian,” “Native American,” “Ashkenazi Jewish,” “Mediterranean,” “Middle Eastern,” and “Other.”

Mentions of race/ethnicity varied significantly by question location (*p* < 0.0001; Fig. 1). Asian race had 6.40 times greater odds of occurring in the answer explanation only relative to the question stem only (95% CI 1.19–34.49; *p* < 0.031). White race had about 90% lower odds of occurring in the answer explanation only (OR 0.09; 95% CI 0.03–0.27; *p* < 0.001) and in both the question stem and the answer explanation (OR 0.10; 95% CI 0.03–0.39; *p* < 0.001; data not shown) relative to the question stem only. Relative to presence in both the question stem and the answer explanation, White race had 9.88 times greater odds of occurring only in the question stem (95% CI 2.56–38.08; *p* < 0.001).

In 10 of 17 examples in which immigration status served as a “clue” for disease, the area to which an infectious disease was described as endemic was a specific country or region; however, for the other seven, the endemic area superseded continental boundaries, often including large areas of the globe (e.g., “Latin America, Africa, or Asia”). The phrase “developing country/ies” or “developing world” occurred six times in reference to endemic areas of infectious disease.

**Figure 2:**
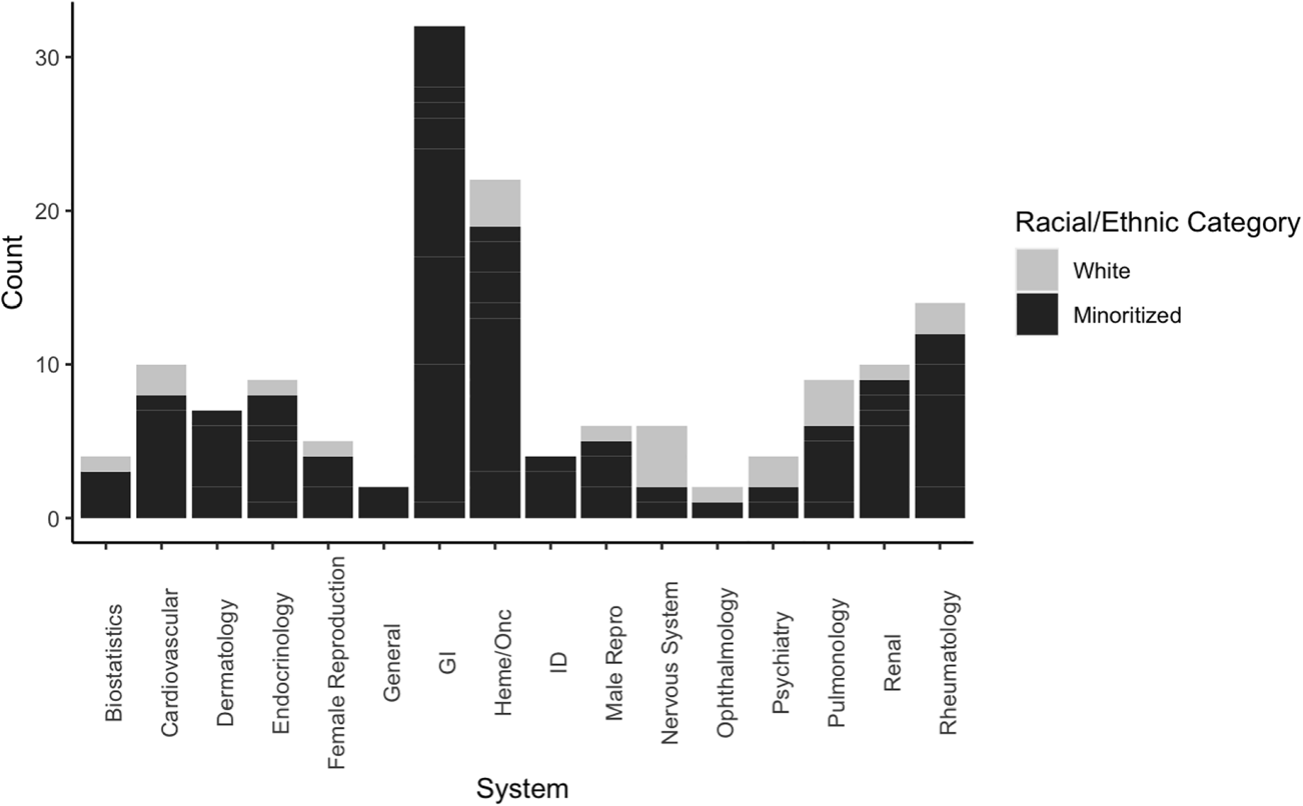
Race/ethnicity by system. This stacked bar plot represents racial/ethnic categories by system. Racial/ethnic categories are represented as counts. Total counts for each system are as follows: Biostatistics (*n* = 4), Cardiovascular (*n* = 10), Dermatology (*n* = 7), Endocrinology (*n* = 9), Female Reproduction (*n* = 5), General (*n* = 2), Gastrointestinal (GI) (*n* = 32), Hematology/Oncology (Heme/Onc) (*n* = 22), Infectious Disease (ID) (*n* = 4), Male Reproduction (Male Repro) (*n* = 6), Nervous System (*n* = 6), Ophthalmology (*n* = 2), Psychiatry (*n* = 4), Pulmonology (*n* = 9), Renal (*n* = 10), Rheumatology/Orthopedics/Sports Medicine (Rheumatology) (*n* = 14). “Minoritized” includes “Black,” “Latinx,” “Asian,” “Native American,” “Ashkenazi Jewish,” “Mediterranean,” “Middle Eastern,” and “Other.”

Mentions of race/ethnicity varied significantly by UWorld system (*p* < 0.0001; Fig. 2). Race/ethnicity occurred most often in the gastrointestinal (GI) system (*n* = 32), hematology/oncology (*n* = 22), and rheumatology/orthopedics/sports medicine systems (*n* = 14).

### Associations Between Mentions of Race, Ethnicity, or Immigration Status and Answer Choice

We found no significant relationship between mentions of White vs. non-White race in the question stem and the percentage of users who selected the most common answer choice. Likewise, we found no significant relationship between immigration region of origin and most common answer choice percentage. We found no significant relationship between whether we coded signifiers in a question stem as “clues,” “distractions,” or “descriptive” and the percentage of users who chose the correct answer choice. Race or ethnicity was frequently coded as “clue,” intended to guide the QBank user toward the correct answer choice, or “distraction,” aimed to guide the user toward an incorrect answer choice. Among questions in which we coded race/ethnicity as “descriptive,” or unrelated to the answer choices or explanation, the patient was described as White/“Caucasian” 72.7% of the time.

### Associations Between Disease Conditions and Racial, Ethnic, and Immigration Categories

Table 2 provides brief definitions, frequencies, and representative examples for each code. Answer explanations frequently identified disease conditions as “more common in,” “associated with,” and “highly prevalent in” specific racial or ethnic groups. Notably, all of these explanations lacked socio-structural context. Twelve questions specifically associated disease conditions with “ancestry” or “descent,” including links between Mediterranean ancestry and beta-thalassemia and between ulcerative colitis and Ashkenazi Jewish descent. Ten questions furthered these epidemiological associations by stating that members of particular racial groups are at increased “risk” for disease conditions. In 18 instances, the racial or ethnic differences in risk were attributed to genetic causes (e.g., *APOL1* associations with kidney disease in Black patients, associations between sickle cell trait and sub-Saharan African ancestry).

**Table 2:** Qualitative Code Definitions, Frequencies, Percentages, and Representative Examples (*N* = 123)

	Brief definition	Frequency (%)	Representative example
Clue	Race or immigration status is mentioned in the question stem to guide the user toward the correct answer choice.	55 (44.7)	A patient with exertional dyspnea is described as Black to suggest a diagnosis of longstanding hypertension.
Distraction	Race or immigration status is mentioned in the question stem to guide the user toward an incorrect answer choice.	15 (12.2)	A Black patient with cough, fatigue, and shortness of breath is initially treated with high-dose corticosteroids for sarcoidosis, but this treatment actually exacerbates the histoplasmosis infection that is causing his symptoms. The answer explanation indicates that sarcoidosis is a reasonable diagnosis given the patient’s symptoms and race but notes that histoplasmosis is the most likely diagnosis.
Descriptive	Race or immigration status is included in the question stem with no clear rationale or link to the answer choices or explanation.	22 (17.9)	A patient with shortness of breath is described as African American and all answer choices relate to malignancies that have not been previously linked to Black race.
Race and ethnicity			
White norm	White/“Caucasian” race is used specifically in the question stem without clear rationale or link to the answer choices or explanation.	16 (13.0)	Descriptions include a 64-year-old White female with nausea and a 35-year-old “Caucasian” male with aplastic anemia.
Epidemiology	Racial differences in disease prevalence.	41 (32.5)	Systemic lupus erythematosus is described as occurring most commonly in African American, Hispanic, and Asian women.
Ancestry	References to “ancestry” or “descent.”	12 (9.8)	Links are made between Mediterranean ancestry and beta-thalassemia and between ulcerative colitis and Ashkenazi Jewish descent.
Racial differences in risk	Race is treated as a risk factor for a disease condition.	11 (8.1)	Black race is listed as a risk factor for prostate cancer in a chart comparing prostate cancer and benign prostatic hyperplasia.
Racial genetics	Associations with racial groups and genetic risks for disease.	9 (7.3)	Increased prevalence of kidney disease in Black patients is linked to variants in the *APOL1* gene.
Race-based management	Descriptions of variations in treatment (or lack of treatment) based on a patient's race.	8 (6.5)	Sodium restriction is described as more effective in Black patients.
Socio-structural	Mention of a patient’s race is contextualized within their social or structural support system or vulnerability.	1 (0.8)	An immigrant patient is described as having few social supports.
Immigration status			
Endemic	Migration from a specific area (e.g., country or region) in which an infectious disease is endemic	10 (8.1)	An immigrant’s prior residence in Southeast Asia, a region where tuberculosis is endemic, suggests a diagnosis of miliary tuberculosis.
Global	Descriptions of endemic areas that supersede continental boundaries (e.g., “Latin America, Asia, and Africa”), generally referring to a global population that excludes Europe and North America.	7 (5.7)	Mitral stenosis is described as a common cause of rheumatic heart disease among patients who have migrated from Latin America, Africa, or Asia.
Developing country	Specific use of the phrase “developing country/ies” or “developing world.”	6 (4.8)	Tuberculosis is described as a common cause of constrictive pericarditis in “developing countries.”
Immigrant–inadequate care	Mentions of immigrants from other countries and absence of prior care or vaccination.	7 (5.6)	A question featuring a 26-year-old pregnant woman mentions that she immigrated to the USA 6 years ago and includes titer information signifying her lack of immunization for measles, mumps, and rubella.
Cultural/behavioral	Attributions of disease risk to cultural practices, beliefs, or behaviors.	7 (5.6)	Migration from North Africa is linked to niacin deficiency due to presumed primary subsistence on corn products
Total		226 segments (123 questions)	

^i^Full code definitions are available in Supplemental Materials

^ii^Frequency, or count, of code and percent representation of all questions in QBank data. Note that the numerators for the percent representation values may differ from the frequency value due to potential code repetition within questions

Migration frequently signaled lack of prior medical care or routine vaccinations; specific dietary behaviors, cultural practices, and beliefs; and infectious disease risk. As with race and ethnicity, references to immigration status often suggested a disease condition other than the one associated with the correct answer choice.

### Race-, Ethnicity-, or Migration-Based Care

In rare instances, answer explanations provided explicit recommendations for alternate care based on race. These include references to sodium restriction for Black patients and use of the Pooled Cohort Equations (also known as the Atherosclerotic Cardiovascular Disease or ASCVD Risk Calculator), which adjusts for race, and earlier diabetes screening in Black and Native Americans. We did not observe overt guidance for tailored management by ethnicity or immigration status, though these were often implied in answer explanations.

## CONCLUSIONS

Our analysis reveals race-, ethnicity-, and migration-based disease associations in the UWorld QBank. These associations have potential for systematic harm, including promoting inaccurate race-based associations and upholding cultural conventions of White bodies as normative. Here, we discuss the implications of three key findings: (1) the low frequency yet unequal distribution of race, ethnicity, and immigration status; (2) the associations with disease conditions in minoritized racial and ethnic groups; and (3) the misrepresentation of immigration status as a risk factor for disease.

### Low Frequency yet Unequal Distribution of Mentions of Race, Ethnicity, and Immigration Status

Mentions of race, ethnicity, and immigration status occurred infrequently, representing only 3.5% of the questions we examined. This contrasts with prior research analyzing the UWorld Step 1 QBank, which found that mentions of race and ethnicity occurred in 20.6% of questions.^13^ This finding may reflect differences between USMLE Step 1 and Step 2 CK or conscious efforts to remove mentions of race and ethnicity from the exam. In our analysis, non-White race and specific ethnic identifiers (e.g., Mediterranean, Ashkenazi Jewish, Middle Eastern) occurred much more frequently than mentions of White race. The invisibility of race and ethnicity (and immigration status) risks promoting color-blind racism and White supremacy due to hegemonic societal conventions of White, cisgender bodies as unmarked.^14,15^ In other words, to describe a 45-year-old woman with fatigue and muscle weakness is to imagine a 45-year-old White, cisgender woman with these symptoms. We therefore discourage the removal of race and ethnicity from the QBank and instead emphasize the need for racial and ethnic categories to remain descriptive rather than to suggest disease risk or guide management.

Mentions of race/ethnicity varied significantly by UWorld system, indicating that the embeddedness of race-based associations may differ among specialties. Recent discussions of race-based medicine describe harmful practices in cardiology, nephrology, obstetrics, and oncology.^3,4^ This suggests that race-based associations with disease conditions and management may be more pervasive in certain specialties relative to others. Accordingly, interventions to reform the use of race, ethnicity, and immigration status in UWorld may yield greater benefit if targeted toward test question contributors from specific specialties. Rather than using race, ethnicity, or immigration status as proxy for genetics, healthcare status, infectious disease exposures, or behavior, individualized details (e.g., family history, immunization status, diet) should be included by specialists and question authors to help students develop a more inclusive and holistic approach to medical care.

### Associations with Disease Conditions in Minoritized Racial and Ethnic Groups

White race had nearly 10 times greater odds of occurring in the question stem only relative to presence in both the question stem and answer explanation. This indicates that race-based associations with disease are less likely to be made with patients of White race. Our qualitative analysis echoes this finding: mentions of White race without clear rationale or link to the answer choices or explanation emerged 16 times.

By contrast, Asian race had 6.4 times greater odds of occurring in the answer explanation only relative to the question stem only. Although the odds ratios for Black and Latinx were not significant, our qualitative analyses suggest that race-based associations are evident across minoritized racial groups. These included epidemiologic associations and descriptions of increased disease risk within racial/ethnic/immigrant groups (Table 2). By presenting these associations without socio-structural context, these diseases are implied to be biological in nature. This is further codified through geneticization, or the attribution of “social, behavioral, or physiological problems” to genetic variation by race.^16^ These models build on descriptive analyses in the UWorld Step 1 QBank,^13^ quantifying the tight associations between minoritized racial groups and disease conditions.

Although recent, individual ancestry may be genetically meaningful when traced to a narrowly circumscribed geographic population of origin, clinicians and researchers alike frequently elide the concepts of ancestry and race through continental descriptors (e.g., “African ancestry”) that lack precision.^17,18^ Mentions of “ancestry” in our analysis commonly corresponded to broad geographic areas (e.g., “Asian,” “Northern European”). The persistence of race and continental ancestry as ready substitutes for more explicit bio- or genetic markers of disease demonstrates how assumptions about the intrinsic, biological nature of race remain unchecked in biomedicine.

Our analyses of the “race-based management” and “distraction” codes demonstrate how race-based associations contribute to racially tailored care and may even potentially lead to harm. In one question, a Black patient with histoplasmosis is initially suspected of sarcoidosis and mismanaged with high-dose corticosteroids, which exacerbates his condition. Here, the test authors tacitly underscore the risks of race-based associations, noting how inappropriately narrow differential diagnoses can lead to iatrogenic harm.

### Misrepresentation of Immigration Status as a Risk Factor for Disease

Seven answer explanations involving immigration status described endemic regions beyond continental boundaries, often including large areas of the globe outside of Europe and North America. The term “developing country” occurred in six answer explanations as an indication of risk for conditions such as rheumatic fever and echinococcus. Infectious disease risk varies within regions, countries, and across continents. Although “developing country” may imply conditions of limited public health infrastructure, these vary widely by geographic and socioeconomic position and cannot be inferred by an individual’s country or continent of origin. This may promote harmful biases against immigrants from low- and middle-income countries, particularly those racialized as Black or Brown.

Among references to immigration status, nearly half discussed migration from areas in which specific infectious diseases are endemic. Many immigrant source countries face high burdens of infectious disease, largely relating to environmental and socioeconomic conditions.^19,20^ The Global Fund for tuberculosis, HIV, and malaria highlights this: these diseases are largely due to limitations of public health infrastructure and subtropical environments.^21^ In these cases, migration history may serve as an important indicator of risk.

However, our analysis of the “distraction” code suggests that immigration status was often used to imply disease risk in a misleading way. This exemplifies a key pitfall of conflating immigration status with ethnicity,^22^ and of using ethnicity (or race) as a proxy for genetic background. Migration from a malaria-endemic region may suggest an individual has an increased likelihood of carrying traits for sickle cell disease or thalassemia; however, careful family history should inform pre-test probability rather than migration history or ethnicity alone.

A robust literature exists on migration as a social determinant of health.^23–26^ This research examines the effects of healthcare policy, authorization status, and language barriers on immigrant health. Across these structural dimensions, immigration status is a real predictor of health outcomes and should affect management decisions; however, our analysis did not reflect this complexity, instead linking immigrants with disease and adverse health beliefs and behaviors. QBanks and multiple choice examinations may not be a fitting modality through which to teach and assess structural competency or respond to the institutions and social conditions that affect health.^27^

Our analysis suggests that immigration status was often used to inappropriately suggest inadequate health maintenance, behavior, culture, and ethnicity; in these instances, appropriate management involved vaccination, nutritional supplementation, or antibiotic treatment. Assumption that immigrants, solely due to their immigration status, have not received routine health maintenance or are not up-to-date with immunizations may contribute to harmful stereotypes of immigrants as “victims” or as being unfamiliar with healthcare routines.^28,29^ Immigration status cannot be used as a proxy for behavior: culture is not static, nations are pluralistic, and individuals are intersectional.^30,31^ For instance, information about high intake of corn or nitroso compounds could be obtained through a careful dietary history of a patient suspected of niacin deficiency or gastric cancer, respectively.

### Limitations

Our study carries limitations. First, we limited our analysis to one study tool for the USMLE Step 2. We recognize that medical students use a variety of study materials and future research might analyze the use of race, ethnicity, and immigration status in other such tools. Second, we were only able to examine the questions available during the capture period of May 28 through August 11, 2020. Repeated analysis as questions update may yield new findings. Third, copyright restrictions prevented direct use of QBank quotes, limiting the specificity of our qualitative analysis. Fourth, although our mixed-methods analyses allow us to draw inferences regarding the function of mentions of race and ethnicity, we are unable to speak to the motivations of the QBank developers for including race, ethnicity, and immigration status. Finally, our analysis of this QBank does not represent the USMLE Step 2 CK exam, although the QBank claims to closely approximate the USMLE.^9^ Despite these limitations, we provide a robust, systematic analysis of the use of race and ethnicity in the UWorld Step 2 QBank.

Our study identifies several problematic associations between racial, ethnic, and immigration categories and disease conditions in the UWorld Step 2 CK QBank. We emphasize the need to account for individual patient details (e.g., family history, immunization status, diet) rather than using race, ethnicity, or immigration status as proxy for genetics, healthcare status, infectious disease exposures, or behavior. Reform of race-based medicine in medical licensing educational resources—and in medical education more broadly—can contribute to health equity.

## Supplementary Information

ESM 1 (DOCX 20 kb)
