# Supplementary material for: Race, Ethnicity, and Immigration Status in a Medical Licensing Educational Resource: a Systematic, Mixed-Methods Analysis
Source: J Gen Intern Med. 2021 May 13;37(5):1045–51. doi: 10.1007/s11606-021-06843-0 (PMC8971213; doi:10.1007/s11606-021-06843-0)
Supplement: Supplementary file 1 — (DOCX 20 kb) [file 11606_2021_6843_MOESM1_ESM.docx]

**Appendix**

# Code System

| 1 ancestry |
| --- |
| 2 immigrants inadequate care |
| 3 racial genetics |
| 4 racial differences in risk |
| 5 cultural/behavioral |
| 6 epidemiology |
| 7 White norm |
| 8 distraction |
| 9 clue |
| 10 endemic |
| 10.1 global |
| 11 developing country |
| 12 race-based management |
| 13 sociostructural |
| 14 superfluous |

# Code Definitions

## 1 ancestry

This code should be used with references to "ancestry" or "descent" (e.g., "Middle Eastern ancestry").

## 2 immigrants inadequate care

This code refers to mentions of (im)migrants from other countries and absence of prior care or vaccination.

INCLUDES:

- presumption of non-vaccinated status, incomplete vaccination schedule, or absence of routine healthcare

- mention of immigrant in addition to mention of non-vaccinated status or absence of routine healthcare

- mention of immigrant in addition to mention of up-to-date healthcare in question stem

EXCLUDES:

- no prior history due to adoption

## 3 racial genetics

This code refers to associations with racial groups and genetic risks for disease (e.g., APOL1 variants in kidney disease).

INCLUDES:

- "risk increased in this population possibly due to genetic predisposition"

- "possibly due to APOL1 variants"

- "Sickle cell hemoglobin mutations... Sub-saharan African..."

- mention of race with genetic inheritance pattern (e.g., autosomal dominant, X-linked)

## 4 racial differences in risk

This code should be used when race is treated as a risk factor for a disease condition.

INCLUDES:

- listing of race as a risk factor for a disease in the answer explanation

- mention of racial group paired with phrase "at risk" or "at increased/greater/less/greatest risk"

- "common cause" of condition in racial group

EXCLUDES:

- mention of racial group paired with phrase "more common in"

## 5 cultural/behavioral

This code refers to attributions of disease risk to cultural practices, beliefs, or behaviors.

INCLUDES:

- culture-bound syndromes

- dietary practices

- consuming contaminated water

## 6 epidemiology

This code refers to racial differences in disease prevalence.

INCLUDES:

- X disease is "more/most common" in Y racial group

- X disease is "associated" with Y racial group

- "high prevalence" in X population

- the first group reported X disease much more frequently for Y patients than the second group

- X is the most likely diagnosis in a patient of Y race

- X disease in more common in Y and Z regions

- clues to X diagnosis include Y race/ethnicity

## 7 White norm

This code should be used specifically when White/Caucasian race is used in the question stem without clear rationale or link to the answer choices or explanation.

NOTE: Even if external data suggests that condition is more prevalent in White populations, if there is no evidence of this provided in the QBank (i.e., in answer explanations or in linked articles), mention of White race is presumed to be superfluous.

## 8 distraction

This code should be used when race or immigration status is mentioned in the question stem to guide the user toward an incorrect answer choice.

## 9 clue

This code should be used when race or immigration status is mentioned in the question stem to guide the user toward the correct answer choice.

## 10 endemic

This code refers to migration from a specific area (e.g., country or region) in which an infectious disease is endemic.

INCLUDES:

- mentions of migration status in question stem

- descriptions of endemic areas in answer explanation

### 10.1 global

This code refers to descriptions of endemic areas that supercede continental boundaries (e.g., "Latin America, Asia, and Africa"), generally referring to a global population that excludes Europe and North America.

## 11 developing country

This code refers to specific use of the phrase "developing country/ies" or "developing world."

## 12 race-based management

This code should be used when variations in treatment (or lack of treatment) based on a patient's race are described.

INCLUDES:

- Pooled Cohort Equations cardiovascular risk calculator

- hypertension management

- diabetes screening

## 13 sociostructural

This code should be used when mention of a patient's race is contextualized within their social or structural support system or vulnerability.

INCLUDES:

- mentions of "social support"

## 14 descriptive

This code should be used when race or immigration status is included in the question stem with no clear rationale or link to the answer choices or explanation.
